# Rapid and ultra-sensitive quantitation of disease-associated α-synuclein seeds in brain and cerebrospinal fluid by αSyn RT-QuIC

**DOI:** 10.1186/s40478-018-0508-2

**Published:** 2018-02-09

**Authors:** Bradley R. Groveman, Christina D. Orrù, Andrew G. Hughson, Lynne D. Raymond, Gianluigi Zanusso, Bernardino Ghetti, Katrina J. Campbell, Jiri Safar, Douglas Galasko, Byron Caughey

**Affiliations:** 1 Laboratory of Persistent Viral Diseases, Rocky Mountain Laboratories, National Institute of Allergy and Infectious Diseases, National Institutes of Health, Hamilton, MT USA; 2 Department of Neurosciences, Biomedicine and Movement Sciences, University of Verona, Verona, Italy; 3 Indiana University School of Medicine, Indianapolis, IN USA; 4 Department of Pathology, Case Western Reserve University School of Medicine, Cleveland, OH USA; 5 Department of Neurosciences, University of California-San Diego, La Jolla, CA USA

**Keywords:** Parkinson, Lewy body, Alzheimer, Diagnosis, Synuclein, Amplification, Cerebrospinal fluid, PMCA, RT-QuIC, Prion

## Abstract

The diagnosis and treatment of synucleinopathies such as Parkinson disease and dementia with Lewy bodies would be aided by the availability of assays for the pathogenic disease-associated forms of α-synuclein (αSyn^D^) that are sufficiently sensitive, specific, and practical for analysis of accessible diagnostic specimens. Two recent αSyn^D^ seed amplification tests have provided the first prototypes for ultrasensitive and specific detection of αSyn^D^ in patients’ cerebrospinal fluid. These prototypic assays require 5–13 days to perform. Here, we describe an improved α-synuclein real time quaking-induced conversion (αSyn RT-QuIC) assay that has similar sensitivity and specificity to the prior assays, but can be performed in 1–2 days with quantitation. Blinded analysis of cerebrospinal fluid from 29 synucleinopathy cases [12 Parkinson’s and 17 dementia with Lewy bodies] and 31 non-synucleinopathy controls, including 16 Alzheimer’s cases, yielded 93% diagnostic sensitivity and 100% specificity for this test so far. End-point dilution analyses allowed quantitation of relative amounts of αSyn^D^ seeding activity in cerebrospinal fluid samples, and detection in as little as 0.2 μL. These results confirm that αSyn^D^ seeding activity is present in cerebrospinal fluid. We also demonstrate that it can be rapidly detected, and quantitated, even in early symptomatic stages of synucleinopathy.

**Electronic supplementary material:**

The online version of this article (10.1186/s40478-018-0508-2) contains supplementary material, which is available to authorized users.

## Introduction

Many neurodegenerative diseases are related to the accumulation of specific misfolded proteins. These deposits are identified upon *post-mortem* analysis of brain tissue, allowing definite diagnoses to be made based on specific neuropathological and molecular findings. Less definitive *intra vitam* diagnoses can be proffered based on specific clinical signs, tissue imaging data, pathological examination of peripheral biopsies, and less-than-specific biomarker levels in the CSF. In particular, early diagnosis can be difficult and discrimination between diseases can be complicated by clinical variability and overlaps in clinical features.

Parkinson’s disease (PD), multiple system atrophy (MSA), dementia with Lewy bodies (DLB) [or Lewy body dementia] are called α-synucleinopathies due to the abnormal accumulation of aggregates of a protein called α-synuclein (αSyn) in the brain. Although the clinical diagnosis of parkinsonism can be relatively simple, the specific diagnosis of PD, especially at early stages, can be difficult. Adler et al. noted that in patients with possible PD (never treated or not clearly responsive to L-dopa) only 26% had autopsy confirmation as PD, while in probable PD (responsive to medications) the diagnostic accuracy was 82% [1]. In DLB, clinical diagnostic criteria for probable DLB predict αSyn pathology with sensitivity of about 80% [18] but early diagnosis of DLB is less accurate due to the overlapping symptoms with other types of dementia. In addition, in 15–20% of patients with Alzheimer disease (AD) at autopsy, concomitant DLB pathology can be found, with only a minority of patients having exhibited clear diagnostic features of DLB [20, 34]. However, in patients with AD and diffuse Lewy body pathology, disease duration was shortened [11], indicating that DLB pathology contributes to dementia progression.

Some pertinent tests indirectly measure the effect of α-Syn pathology (e.g., dopamine receptor SPECT or PET scans, and MIBG cardiac scintigraphy), while the sensitivity and specificity of skin, salivary gland and colonic biopsy for PD or DLB has not been established in large scale studies. In these clinical settings of PD and DLB, the presence of a biomarker that indicates that abnormal pathological forms of a αSyn are present would improve diagnostic accuracy not only for prognostic purposes but also for cohort selection in disease-modifying clinical trials for PD. Attempts to determine if cerebrospinal fluid (CSF) levels of total, phosphorylated or oligomeric a-syn are diagnostically useful have been variable and controversial between studies [reviewed in [27]], and the diagnostic utility of immunoassays for these forms of αSyn in CSF remains unclear [21, 31].

However, two recent studies have provided evidence that analysis of a distinct feature of disease-associated forms of αSyn (hereafter abbreviated αSyn^D^), namely their amyloid seeding activity, may have substantial diagnostic utility for PD and DLB [7, 35]. The rationale for the seeding activity assays is that the αSyn^D^ deposits contain fibrils, or subfibrillar oligomers, that propagate by a seeded polymerization mechanism in which αSyn^D^ templates, or seeds, conversion of non-fibrillar αSyn into larger oligomeric or aggregate, fibrillar forms. Mechanistically similar assays called Real-Time Quaking-Induced Conversion (RT-QuIC) have provided ultrasensitive, specific and quantitative diagnostic tests for prion diseases [2, 39]. RT-QuIC assays are multi-well plate-based reactions that can rapidly amplify oligomeric/multimeric prion seeds by as much as a trillion-fold [8, 24, 26, 39]. Prion RT-QuIC assays have been applied successfully to a variety of biological samples including brain [29, 39, 41], cerebrospinal fluid (CSF) [2, 5, 17, 24, 33], whole blood, plasma [26, 38], urine [14], and nasal brushings [23, 40]. They are being widely implemented for the diagnosis of prion diseases in humans and animals. Notably, our recent studies demonstrated provisional 100% diagnostic sensitivity and specificity in diagnosing human sporadic Creutzfeldt-Jakob disease using CSF and/or nasal swabs [4].

Green and colleagues adapted the RT-QuIC approach to synucleinopathies and applied it to a total of 137 PD and DLB cases and controls [7]. Their assay (αSyn RT-QuIC) has given 95 and 92% sensitivity for PD and DLB patients, respectively, with 100% specificity. Soto and colleagues developed a similar assay called αSyn protein misfolding cyclic amplification (αSyn-PMCA) which gave 89% sensitivity for PD and 97% specificity in analyses of 173 total cases and controls [35]. In these assays, 5–40 μl aliquots of CSF are added to reactions containing recombinant αSyn (rαSyn). Any αSyn^D^ seeds in the sample initiate amyloid fibril formation by the recombinant αSyn which, in turn, enhances the fluorescence of thioflavin T (ThT). The reactions are performed over ~ 5 [7] to 13 days [35]. Sano and colleagues have described an αSyn RT-QuIC assay that detects DLB αSyn^D^ seeding activity in brain tissue at extreme dilutions in < 4 days [32]. Bernis and colleagues showed that 10% brain homogenate samples from mice inoculated with human MSA or incidental Lewy body disease brain tissue could seed fibrillization of rαSyn in 1–2 days [3]. Here we report that by using a mutant rαSyn substrate and optimized reaction conditions, αSyn RT-QuIC assays on CSF specimens can be completed within 1–2 days with high diagnostic sensitivity and specificity.

## Materials and methods

### Clinical assessment

All subjects provided consent to clinical assessment, including longitudinal follow-up, and to lumbar puncture to obtain CSF, under UCSD IRB-approved protocol #080012. All procedures performed in this study were in accordance with the 1964 Helsinki declaration and its later amendments or comparable ethical standards. Some subjects died during the follow-up period, and had consented to their brains being obtained at autopsy.

All subjects underwent a detailed clinical research assessment, including review of outside medical records, history of cognitive and motor symptoms, mental state examination with the Mini-Mental State Exam or Montreal Cognitive Assessment, and detailed neuropsychological testing, structured physical neurological examination, including the Unified Parkinson’s Disease Rating Scale (UPDRS) Part III motor examination. All subjects were enrolled in a research protocol that allowed annual follow-up reassessment and received at least one follow-up assessment after their baseline visit. Neuroimaging (MRI and in some instances FDG PET scan or DaTscan) results were reviewed when available. The research diagnoses were made by consensus of two neurologists who reviewed all of the available clinical information. Research diagnoses followed published criteria: controls had no history of major neurological or psychiatric illness and were normal on cognition and neurological examination; patients with AD met criteria for probable AD (NIA-AA 2011). For PD, criteria proposed by the Movement Disorder Society were used [30], and research guidelines were applied to diagnose PD-MCI [16], PD-dementia and DLB (possible and probable DLB were diagnosed according to McKeith [19]).

### Lumbar puncture and CSF handling

Lumbar punctures (LPs) were performed in the early morning, after a fast of at least 8 h. Subjects were either sitting or lying, and LPs were performed with sterile technique using an atraumatic needle. CSF (15–20 mL) was withdrawn into a polypropylene tube and a sample was sent for analysis of cell count, total protein and glucose to a local laboratory. The remaining CSF was gently mixed, centrifuged at 1500 g for 10 min, then aliquotted in 500 μL fractions into polypropylene cryotubes, flash frozen and stored at − 80 °C.

### Autopsy brain analysis

Procedures at autopsy at the UCSD Alzheimer’s Disease Research Center are as follows: the brain is divided sagittally and the left hemibrain is fixed in 10% buffered formalin while the right hemibrain is sectioned coronally and then frozen at − 70 °C in sealed plastic bags. Routinely, tissue blocks from the right hemibrain of the midfrontal, inferior parietal, and superior temporal cortices, primary visual cortex in the occipital cortex, hippocampus, basal ganglia, substantia nigra and cerebellum are removed and placed in 2% paraformaldehyde for subsequent thick sectioning by vibratome. Tissue blocks adjacent to the ones described above are stored at − 70 °C for subsequent immunoblot analysis for synaptic proteins and Aβ species (soluble and oligomers). Vibratome sections (40 μm thick) are stored in cryoprotective medium at − 20 °C for subsequent immunochemical studies. The formalin-fixed left hemibrain is serially sectioned in 1 cm slices and tissue blocks from the regions described above are processed for histopathological examination by H&E, and Thioflavin-S (Thio-S) to detect tau and β-amyloid deposits. Lewy body pathology is evaluated using phosphorylated α-synuclein immunoreactivity with a mouse monoclonal antibody at 1:20,000 (BioLegend Cat# 825701 RRID:AB 2564891). Pathological diagnoses of AD and DLB are made using National Institute on Aging-Alzheimer’s Association (NIA-AA) guidelines [22].

### K23Q rαSyn expression vector preparation

DNA sequences coding for human α-synuclein sequence (Accession No. NM_000345.3) amino acid residues 1–140 (wildtype) were amplified and ligated into the pET24 vector with an N-terminal His-tag (EMD Biosciences) and sequences were confirmed. The α-synuclein K23Q mutation [15] was engineered using Q5 Site-Directed Mutagenesis (NEB) using the primers CCACACCCTGTTGGGTTTTCTCAG and CAGAAGCAGCAGGAAAGAC. The plasmids were transformed into BL21(DE3) *Escherichia coli* (EMD Biosciences).

#### rαSyn protein purification

Five ml of LB media containing 50 μg/mL kanamycin were inoculated from a glycerol stock of *E. coli* bacteria containing vectors either for wildtype (WT) or K23Q rαSyn protein expression. Following 4–5-h incubation with continuous 225 rpm agitation at 37 °C, 1 L of the auto-induction media [9] also containing 50 μg/mL kanamycin was prepared and the 5 mL starter culture was added. The cells were grown in a shaking incubator at 37 °C, 225 rpm, overnight. The next day cells were harvested by splitting the 1 L culture into four 250 ml conical tubes and centrifuging at 3273×*g*, 4 °C, 10 min.

Cells were lysed using an osmotic shock protocol modified from Paslawski et al. [28]. Using a 25 mL serological pipette, the cell pellets were gently resuspended in 10% volume of room temperature osmotic shock buffer, (25 mL per 250 mL of cell culture before centrifugation) and incubated at room temperature for 10 min. The suspension was centrifuged at 9000×*g*, 20 °C, 20 min. The supernatant was discarded and the pellet was gently resuspended in 10 mL of ice-cold water per pellet, using a 25 mL serological pipette. The cell suspensions were pooled into two 50 mL tubes to 20 ml each. 20 μL of saturated MgCl_2_ was added to each 20 mL suspension. The suspension was then mixed and incubated on ice with mild rocking for 3 min. Next, the suspension was centrifuged at 9000×*g*, 4 °C, 30 min. The supernatant was collected in a 100 ml glass beaker that contained a stir bar for rapid continuous mixing while being careful not to incorporate air bubbles. The pH was reduced to pH 3.5 by adding a bolus of ~ 800 μl 1 M HCl followed by additional 25 μl increments as necessary while continuously monitoring the pH. A large amount of white precipitate was generated. The suspension was then incubated with gentle stirring at room temperature for 10 min, avoiding the formation of air bubbles. The tubes were centrifuged at 9000×*g*, 4 °C, 30 min, and the supernatant was collected in a fresh 100 ml beaker using continuous agitation with a stir bar. The pH was adjusted to 7.5 with an ~ 800 μl bolus of 1 M NaOH followed by 25 μl increments as necessary. The protein extract was then filtered through a 0.45 μm filter. Next, the extract was loaded onto a 5 ml Ni-NTA column (Qiagen) on an Äkta Pure chromatography system (GE) and washed with 20 mM Tris, pH 7.5 at room temperature. The column was further washed with 50 mM imidazole, 20 mM Tris, pH 7.5 which generated a peak that was not collected. A linear gradient up to 500 mM imidazole in 20 mM Tris, pH 7.5, was performed and a peak was collected between 150 and 375 mM imidazole (Additional file 1). This peak was then loaded onto a Q-HP column (GE) and washed with 20 mM Tris, pH 7.5. The column was further washed with 100 mM NaCl, 20 mM Tris, pH 7.5. A linear gradient up to 500 mM NaCl in 20 mM Tris pH 7.5 was performed and a peak was recovered between 300 and 350 mM NaCl. The protein was filtered through a 0.22 μm filter and dialyzed against water overnight at 4 °C using a 3 kDa MWCO dialysis membrane. The next day, the protein was moved into fresh water for another 4 h dialysis. The protein concentration was determined with a UV–VIS spectrophotometer using a theoretical extinction coefficient at 280 nm of 0.36 (mg/mL)^− 1^ cm^− 1^. The protein was lyophilized in aliquots and stored for a final concentration of ~ 1.0 mg/ml once resuspended in 500 μL of 40 mM phosphate buffer (pH 8.0). These aliquots were stored at − 80 °C until further use.

For comparative studies, human recombinant full-length (1–140 aa) WT αSyn was also purchased from Stratech, (Cambridge, UK).

Endotoxin concentration levels for rαSyn protein purifications was determined using a ToxinSensor Endotoxin Detection kit (GenScript) according to manufacturer’s protocol.

### SDS-PAGE analysis

Samples were prepared in 2× sample loading buffer (final concentration: 62.5 mM Tris–HCl, pH 6.8, 5% glycerol, 3 mM EDTA, 5% SDS, 0.02% bromophenol blue, 4 M urea, 4% β-mercaptoethanol) and boiled for 5 min. Proteins were separated by gel electrophoresis using 10% Bis-TrisNuPAGE gels (Invitrogen). For total protein analysis, gels were stained with GelCode Blue Safe Protein Stain (Thermo Scientific, 24,594) according to the manufacture’s protocol.

#### Preparation of synthetic wildtype rαSyn fibrils

A 100 μL solution of 1 mg/ml WT rαSyn in PBS was subjected to continuous 1000 rpm shaking at 37 °C for 3 days in a 1.5 mL tube. The products were ThT-positive, indicating the presence of amyloid, and the OD_280_ and Western blot comparisons of supernatants before and after centrifugation indicated that > 30% of the total rαSyn was insoluble and therefore aggregated.

#### Brain homogenate preparations

Brain homogenates (BH; 10% *w*/*v*) were prepared by homogenizing the tissue in PBS using a Bead Beater (Biospec Products; 11079110z) for 1 min at maximum speed. The homogenate was then spun at 2000 xg for 2 min at room temperature and the supernatant was transferred to a new tube and stored at − 80 °C for αSyn RT-QuIC analysis. For αSyn RT-QuIC testing, BHs were serially diluted in PBS.

#### αSyn RT-QuIC protocol

**Fig. 1:**
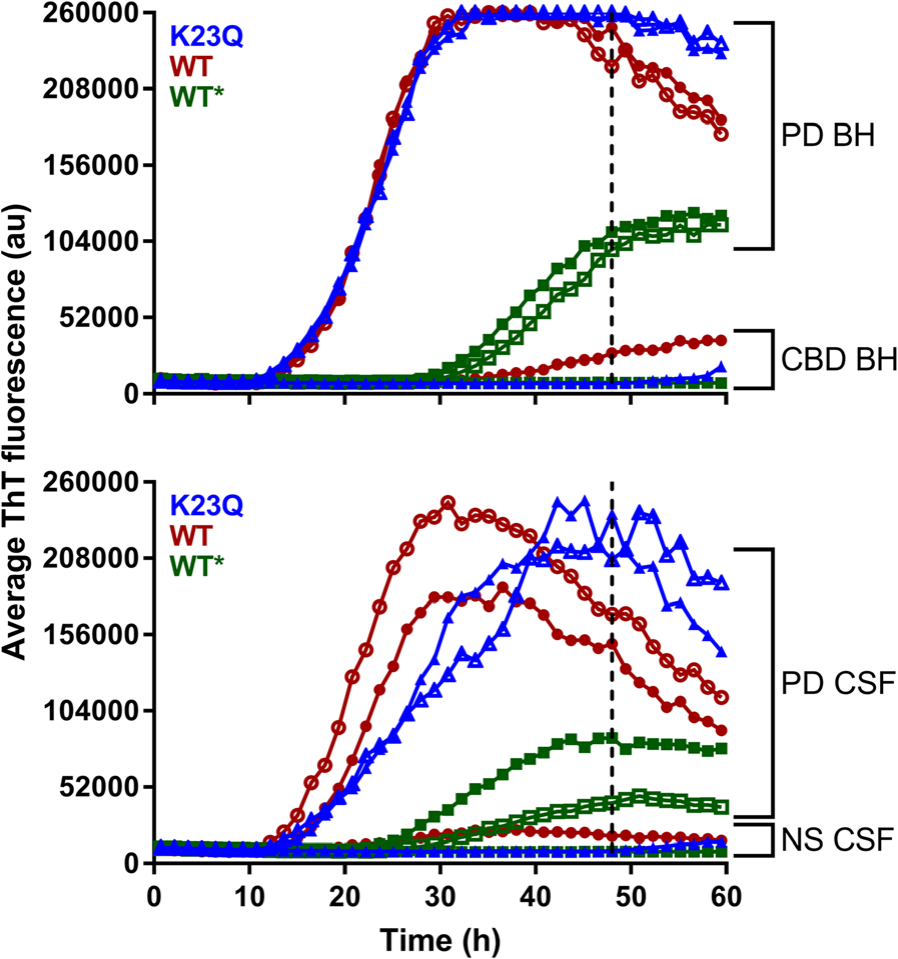
Detection of αSyn seeding activity in BH and CSF using K23Q (blue), WT (red) and WT* (green; commercial wild-type rαSyn lacking a 6× histidine tag [7]) substrates. Reactions were seeded in quadruplicate with brain homogenate (BH) (top panel) or CSF (bottom panel) from Parkinson’s disease (PD) or non-synucleinopathy (NS) including corticobasal degeneration cases (CBD BH) and healthy donors (NS CSF). For the BH assays, 10^− 3^ (closed symbols) or 10^− 4^ (open symbols) brain tissue dilutions from a single PD or NS case were used. For the CSF assays, 15 μl (undiluted) from two PD cases and one NS case was used. Each sample trace represents the average ThT signal of quadruplicate wells. For clarity only every other data point is plotted. The vertical dashed line designates the assay cutoff time used in subsequent analyses

RT-QuIC reactions were performed in black 96-well plates with a clear bottom (Nalgene Nunc International). We preloaded plates with 6 glass or silica beads (1 mm in diameter, BioSpec Products or 0.8 mm, OPS Diagnostics, respectively) per well. Silica beads were ultimately chosen over the glass beads because of some occasional batch to batch variability that was observed with the glass beads. For brain homogenate seeded reactions, 2 μL of indicated BH dilutions were added to wells containing 98 μL of the reaction mix to give final concentrations of 40 mM phosphate buffer (pH 8.0), 170 mM NaCl, 0.1 mg/ml of the designated rαSyn (filtered through a 100 kD MWCO filter immediately prior to use), and 10 μM ThT. The plate was then sealed with a plate sealer film (Nalgene Nunc International) and incubated at 42 °C in a BMG FLUOstar Omega plate reader with cycles of 1 min shaking (400 rpm double orbital) and 1 min rest throughout the indicated incubation time. ThT fluorescence measurements (450 +/− 10 nm excitation and 480 +/− 10 nm emission; bottom read) were taken every 45 min. After the initial testing (data in Fig. 1) the fluorimeter gain settings were adjusted to maintain the fluorescence response within the readable range.

In the case of CSF seeded reactions, wells pre-loaded with 6 glass or silica beads were given  85 μL of a reaction mix adjusted to give final reaction concentrations of 40 mM phosphate buffer (pH 8.0), 170 mM NaCl, 0.1 mg/ml rαSyn, 10 μM thioflavin T (ThT) and 0.0015% sodium dodecyl sulfate (SDS) and then 15 μL CSF, or dilutions thereof in normal pooled CSF. The reaction plates were subjected to shake-rest cycles as for BH samples above and the reactions classified as RT-QuIC-positive or -negative based on criteria similar to those previously described for RT-QuIC analyses of brain specimens [23, 39]. Briefly, a ThT fluorescence threshold was calculated as the average fluorescence for all samples within the first 10 h of incubation, plus three Standard Deviations (SD). A sample was considered positive overall when at least two of four replicate wells crossed this calculated threshold. When only one of the quadruplicates crossed the threshold, the analysis was repeated.

## Results

### Rapid detection of αSyn^D^ by αSyn RT-QuIC

In developing our αSyn RT-QuIC, we focused primarily on a recombinant 6× histidine-tagged K23Q mutant of αSyn as a soluble rαSyn substrate for αSyn^D^-induced fibrillization. Recombinant K23Q αSyn was reported to fibrillize with kinetics similar to wild-type (WT) αSyn when seeded with preformed synthetic WT αSyn fibrils, but was slower to spontaneously fibrillize in the absence of preformed seeds [15]. This latter characteristic, we hypothesized, might improve the sensitivity of an αSyn RT-QuIC assay by enhancing the kinetic distinction between reactions seeded with samples from synucleinopathy cases versus controls. We iteratively optimized the K23Q purification protocol and αSyn RT-QuIC reaction conditions. The best purification protocol to date involves lysis by osmotic shock followed by acid precipitation and sequential metal-ion affinity and ion exchange chromatography steps [28]. No protein impurities were observed by SDS-PAGE analyses of our K23Q mutant rαSyn, our similar preparation of a histidine-tagged WT αSyn, or a commercial wild-type αSyn preparation (without a 6× histidine tag) (WT*) that was used for the previously described αSyn RT-QuIC assay [7] (Additional file 1). However, because lipopolysaccharide (LPS) can contaminate bacterially derived protein preparations and might influence fibrillization, we assayed the three rαSyn preparations and found that whereas our WT and K23Q rαSyn preparations were negative for LPS in this assay (< 0.25 EU/ml), the WT* preparation had ≥ 0.25 EU/ml LPS.

**Fig. 2:**
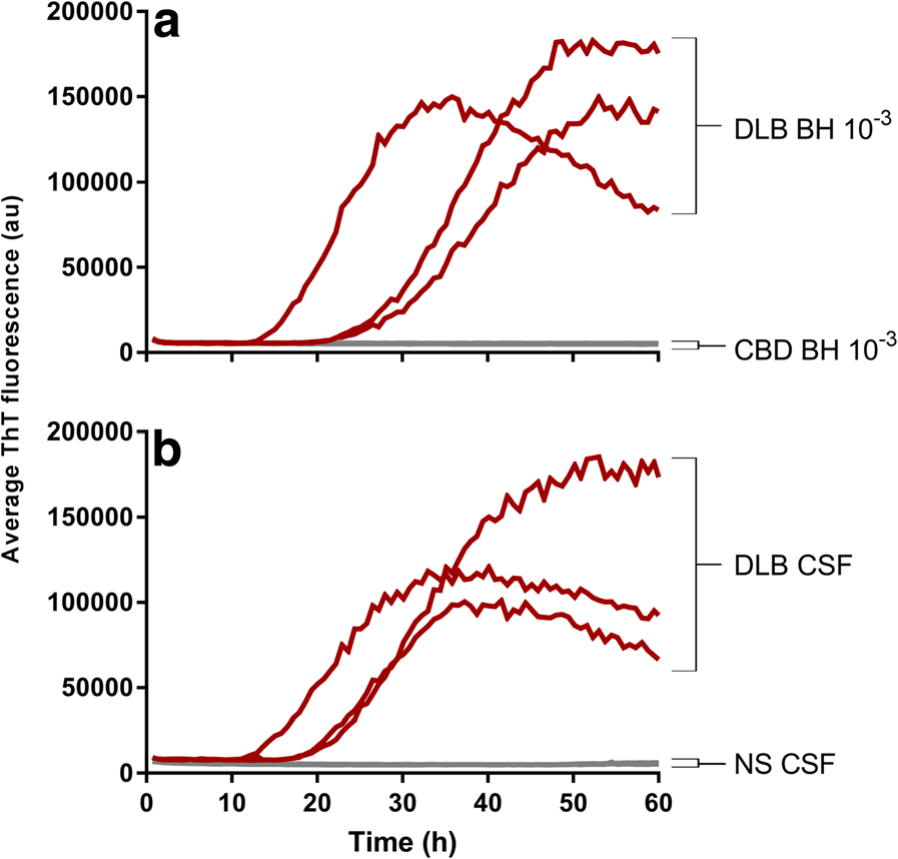
Detection of αSyn seeding activity in BH (**a**) and CSF (**b**) from cases with DLB but not non-synucleinopathy cases using K23Q rαSyn. Two μl of 10^−3^ dilutions of DLB (red; *n* = 3) or CBD (gray; *n* = 3) BH, or 15 μl (undiluted) CSF from DLB (red; *n* = 3) or healthy donors (NS CSF, gray; *n* = 3) were used per reaction. Each trace represents the average ThT signal of the four replicate wells

In the αSyn RT-QuIC assay itself, the sample volume, SDS concentration, temperature, bead size and number were particularly influential in improving the speed, sensitivity and specificity of the αSyn RT-QuIC assay for clinical samples (data not shown). Analyses of brain homogenates (BH) and CSF samples from a small initial set of synucleinopathy (PD and DLB) cases and non-synucleinopathy (NS) cases indicated that, whereas the NS brain and CSF specimens gave no positive RT-QuIC reactions above a threshold fluorescence (see Materials and Methods) over the 48-h reaction period, the PD and DLB samples gave positive responses within ~ 18–35 h for BH and ~ 15–24 h for CSF (Figs. 1 and 2; Additional file 2). When prepared in this way, K23Q (Fig. 1, blue traces) and WT rαSyn (red traces) gave similar responses to seeding with PD brain tissue (10^− 3^–10^− 4^ dilutions; Fig. 1A; Additional file 2) or CSF (15 μl; Fig. 1B; Additional file 2) but the WT rαSyn was more prone to give modest increases in ThT fluorescence in negative control reactions. The WT* rαSyn (green traces), had slower responses and lower maximum ThT fluorescence readings when seeded with PD samples than our WT and K23Q rαSyn substrate preparations. We do not know how the WT* rαSyn was prepared, so either its preparation, its lack of 6× histidine tag, or LPS contamination might be responsible for its weaker responses to seeding compared to our preparations of WT and K23Q rαSyn. With the more rapid PD-seeded reactions with our K23Q or WT rαSyn substrates, we observed decreases in average ThT fluorescence after maximum fluorescence had been achieved. We have observed similar decreases in prion RT-QuIC reactions (e.g. [24]), but their cause has not been determined. Based on these data and the previously published work [15] we have used our K23Q mutant rαSyn preparations in subsequent experiments.

### Blinded analysis of CSF from synucleinopathy cases and controls

We performed blinded analyses of a larger set of CSF specimens obtained *antemortem* from synucleinopathy cases and controls described in Table 1 and Additional file 3. AD cases (*n* = 16) were included as examples of a neurodegenerative protein misfolding disease that usually, but not always [10], lacks synucleinopathy. As such AD cases were analyzed as a separate category from NS samples. Other types of NS samples, including two progressive supranuclear palsy cases and one corticobasal degeneration case, were also included as controls. For many of the synucleinopathy (PD and DLB) cases, the CSF specimens were collected early in the overall clinical course of disease (Additional file 3). For 8 subjects with PD, the CSF was obtained while motor symptoms and signs were still too mild to warrant treatment with L-dopa or another drug with dopaminergic actions (de novo PD). The consensus diagnosis at follow-up or, if available, the autopsy diagnosis was used as the gold standard diagnosis. Final diagnoses of cases and controls based on 1 year or longer of clinical follow up after the LP, and/or autopsy examination of the brain, was thus used as the final diagnosis (Additional file 3). Two cases (13/020 and 14/045 in Additional file 3) were initially diagnosed with AD but with progression had recurring visual hallucinations that are the most robust of the clinical features that predict DLB. Thus, on follow-up, these cases were given diagnoses of possible DLB.

**Table 1:** Demographic data and cognitive impairment at the time of lumbar puncture (LP) in studied subjects

Final diagnosis	n	Age at onset (years)	Age at LP (years)	Mean interval between onset and LP (years)	Sex (M:F)	MMSE^a^
Dementia with Lewy Bodies	17	69.6 ± 7.8	73.8 ± 7.8	4.2	17:2	23.0 ± 4.6
Parkinson’s Disease	12	63.1 ± 12.0	66.0 ± 12.9	2.9	11:1	28.9 ± 1.1
Alzheimer’s Disease	16	69.9 ± 9.1	73.9 ± 9.1	4	12:4	22.9 ± 3.3
Control^b^	12	n/a	71.3 ± 7.0	n/a	4:8	28.8 ± 1.2
Other^b^	3	65.7 ± 11.4	67.7 ± 10.7	2	2:1	20.5 ± 8.1

^a^MMSE: Mini–Mental State Examination, ^b^“controls” and “others” were grouped into “non-synucleinopathies” for analysis

**Fig. 3:**
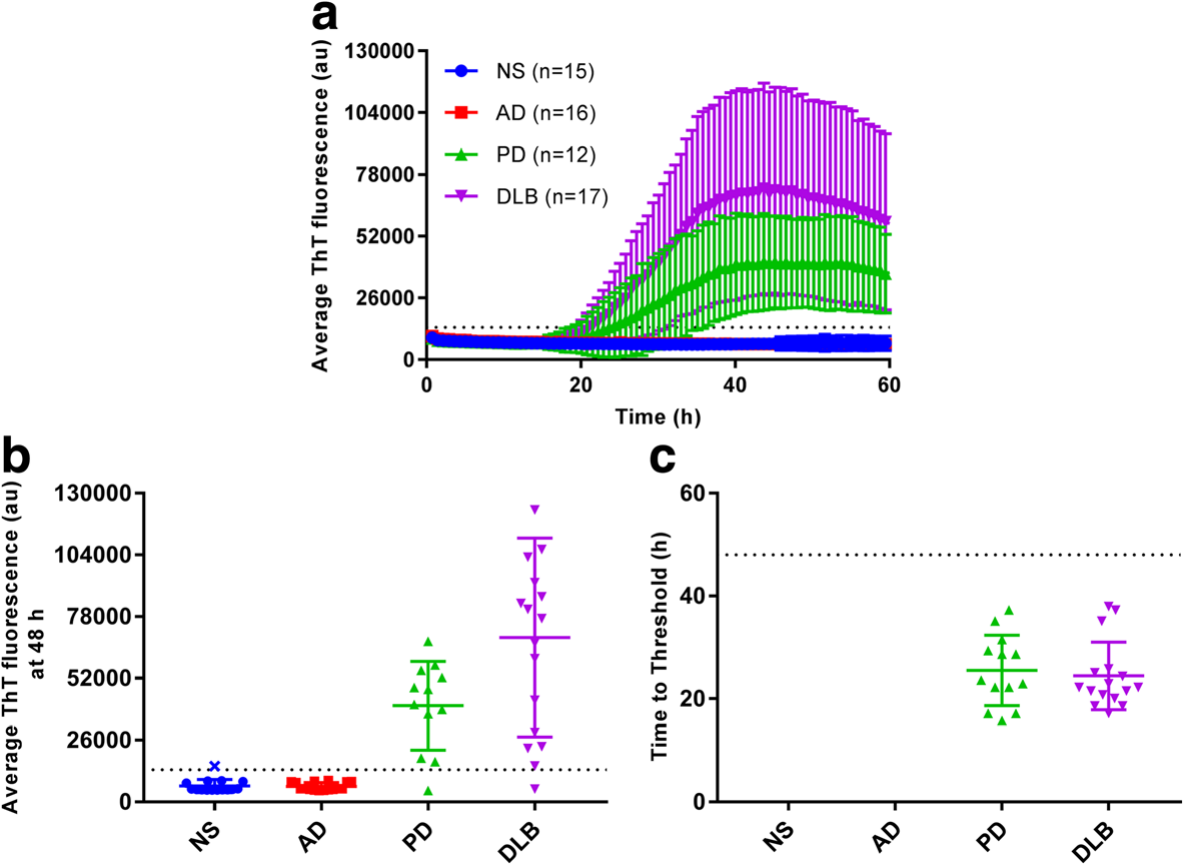
Blinded testing of CSF samples by α-synuclein RT-QuIC. Samples from non-synucleinopathy (NS), Alzheimer’s disease (AD), dementia with Lewy bodies (DLB) or Parkinson’s disease (PD) patients, were tested blinded using the K23Q substrate. Quadruplicate reactions were seeded with 15 μL of CSF. Each sample trace represents the average ThT signal of the four wells. Panel **a** shows the average fluorescence enhancement kinetics for the AD, DLB and PD patients over time along with the associated standard deviation at each time point. Data points in Panel **b** indicate the average fluorescence obtained for each individual case at 48 h. Bars show the average +/− SD for type of case. The dashed line shows the fluorescence threshold for a positive result. Data points in Panel **c** show the hours required for the average fluorescence to exceed the threshold for individual cases. Bars show the average +/− SD for type of case. The dashed line indicates the end of the reaction at 48-h. Blue x symbol indicates sample 15/044 which was tested twice and both times had only one well crossing fluorescence threshold out of the four replicates. This sample was considered negative, as it did not meet our criteria for overall sample positivity (see Materials and Methods)

Almost all of the PD (11/12) and DLB (16/17) CSF samples, including those obtained from the two possible DLB cases, gave positive RT-QuIC responses within 15–35 h (Fig. 3). The average reaction time required to exceed our designated positivity threshold (see Materials and Methods) was similar for the PD and DLB specimens (Fig. 3c). Notably, most of the control cases without any clinical or neuropathological (when available) indication of synucleinopathy were negative in all 4 replicate αSyn RT-QuIC reactions. One case (15/044 in Additional file 3) with a diagnosis of primary progressive aphasia and frontotemporal dementia was positive in 1 of 4 replicate reactions in two independent assays (Fig. 3b; blue “x”). Although it did not meet our criterium of having ≥2 of 4 positive replicate reactions for an overall designation as a positive sample, this case might represent an atypical case of DLB with a clinical diagnosis of primary progressive aphasia [12] with marginally detectable amounts of αSyn^D^ in the CSF. No αSyn^D^ was detected by immunostaining in the midfrontal, inferior parietal, and superior temporal cortices, primary visual cortex in the occipital cortex, hippocampus, basal ganglia, substantia nigra and cerebellum; however, this analysis did not include the amygdala, which can be an initial site of αSyn^D^ accumulation. Thus, while not meeting criteria to be considered positive for αSyn^D^ seed activity, we cannot rule out the presence of some αSyn^D^ pathology in this patient at this time.

Overall, the results from this blinded panel indicated diagnostic sensitivities, i.e. the percentage of cases giving positive RT-QuIC responses, of 93% for both PD and DLB. None of the non-synucleinopathy or AD controls met criteria to be considered positive RT-QuIC responses resulting in an apparent specificity of 100%.

### Relative αSyn seeding activities in CSF and brain tissue from PD and DLB cases

**Fig. 4:**
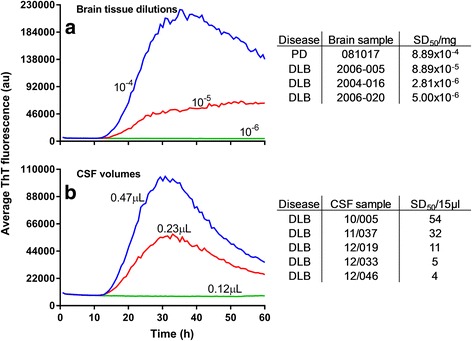
End-point dilutions of synucleinopathy BH (**a**; sample # 081017) or CSF (**b**; sample # 10/005) samples by αSyn RT-QuIC. Each sample trace represents the average ThT signal of quadruplicate wells. Tables to the right of each graph indicate the concentration of SD_50_ units calculated by Spearman-Kärber analysis for these, and additional, cases. End-point dilution experiments used for the additional calculated values shown in the upper and lower panels are provided in Additional files 4 and 5, respectively

To quantify the αSyn RT-QuIC seeding activities in samples from synucleinopathy cases, we performed end-point dilution analyses of frontal cortex brain tissue from representative PD (*n* = 1) and DLB (*n* = 3) cases and CSF samples from 5 DLB cases. All 4 brain samples indicated that positive reactions were obtained out to 10^− 5^–10^− 6^ dilutions of either the PD and DLB tissues (Fig. 4). Positive reactions were obtained from as little as 0.2 μl CSF per reaction well in DLB cases (Fig. 4). Spearman-Kärber analyses [6] provided estimates of the concentrations of seeding activity units giving positive reactions in 50% of replicate reactions, i.e., the 50% “seeding doses” or SD_50_s [39] (Fig. 4). The DLB and PD brain samples contained ~ 10^5^-10^6^ SD_50_ per mg of tissue while the CSF samples had 4–54 SD_50_s per 15 μl, i.e., our usual sample volume. The latter results indicated that these synucleinopathy CSF specimens had seeding activities that are substantially higher than the minimum detectable level of 1 SD_50_. However, on a per weight basis, seeding activity in brain tissue appeared to be 10^4^–10^5^-fold higher than the seeding activities measured in PD and DLB CSF specimens (Fig. 4). We note that slightly different conditions were used for the brain homogenate and CSF specimens because neither of the reaction conditions alone was well suited for detecting seeding activity in both types of samples. These different conditions, in addition to differences in absolute seed concentrations, seed characteristics, or sample matrix components, might have affected the relative seeding activities observed in brain and CSF specimens.

### Analytical sensitivity using synthetic αSyn fibrils

**Fig. 5:**
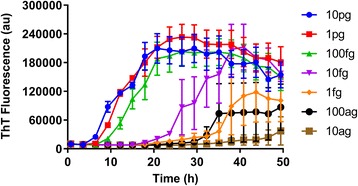
End-point dilutions of synthetic seeds spiked into CSF. Synthetic rαSyn fibrils were generated by continuous shaking at 1000 rpm at 37 °C for 3 days in a 1.5 mL tube containing 100 μL of 1 mg/ml WT rαSyn. Samples were monitored by ThT fluorescence. Following fibrilization the samples were spiked into non-synucleinopathy CSF and diluted in 10-fold serial dilutions. Each sample trace represents the average ± SEM ThT signal of quadruplicate wells. For clarity, data points were plotted every fourth point and negative controls, which were all below the positivity threshold, are not displayed

Finally, to obtain an indication of the analytical sensitivity of our αSyn RT-QuIC, we prepared synthetic rαSyn fibrils, spiked them into non-synucleinopathy CSF and assayed serial dilutions. As little as 100 ag of the synthetic fibril preparations gave at least 2/4 positive replicate reactions (Fig. 5), which was at least as sensitive analytically as the αSyn PMCA assay [35].

## Discussion

The ability to detect αSyn^D^ as a causative pathological biomarker for syncleinopathies has important implications in diagnostics, the development of therapeutics, and fundamental studies of αSyn^D^-based pathogenesis. Recent studies have demonstrated diagnostic utility for αSyn RT-QuIC and closely related αSyn PMCA assays using CSF specimens [7, 35]. Here we present an αSyn RT-QuIC assay with similar diagnostic accuracy but much reduced assay time, i.e. 1–2, rather than 5–13 days. Sano and colleagues detected αSyn seeding activity of DLB brain in 3–4 days [32], but as brain has much higher concentrations of αSyn^D^ seeding activity than CSF (Fig. 4), it is unclear how well their αSyn RT-QuIC assay would perform with CSF specimens. In any case, our reduced assay time markedly enhances the cost effectiveness and practicality of the αSyn RT-QuIC analyses of CSF. Most of the CSF specimens that we analyzed were collected relatively early in the disease course of the given synucleinopathy. The early detection of αSyn^D^ is particularly helpful, firstly, because the accuracy of diagnoses based on other clinical indices is poorest in the earlier phases of disease, and*,* secondly, because the earlier the diagnosis, the earlier that any appropriately targeted therapies can be initiated before further tissue damage is done.

Improvements in the early diagnosis of synucleinopathies should also aid in the selection of suitable patients and controls for therapeutic trials. Furthermore, the ability to serially measure relative levels of αSyn^D^ in treated and untreated cohorts may provide an alternate means of monitoring the effects of treatments, especially those aimed at reducing the burden of αSyn^D^ in the brain. Here we have used end-point dilution analysis for quantitation by αSyn RT-QuIC, an approach that has been helpful in many studies using prion RT-QuIC [23, 24, 39]. A potential alternative approach to quantitation from RT-QuIC assays is the comparison of lag phases or times-to-threshold [13, 36] but further studies will be required to document its utility in αSyn RT-QuIC analyses of various specimen types.

Consistent with previous findings for αSyn PMCA [35], αSyn RT-QuIC is capable of detecting sub-femtogram amounts of rαSyn fibrils that are orders of magnitude less than those required for more conventional assays such as Western blotting and ELISA, with typical detection limits for total levels of αSyn, in the ng and pg range, respectively. The αSyn RT-QuIC, however, detects only the forms of αSyn^D^ capable of seeding further αSyn misfolding. How our αSyn RT-QuIC sensitivity for synthetic rαSyn amyloid seeds relates to its absolute sensitivity for any given form of αSyn^D^ in tissues is difficult to determine accurately because different forms of αSyn^D^ may have different seeding capacities per unit mass. Nonetheless, based on the average kinetics of seeding using PD or DLB CSF in Fig. 3, one might estimate femtogram levels of seeding capable αSyn^D^ may exist in the CSF of PD or DLB patient when compared to the synthetic rαSyn amyloid seeding kinetics in Fig. 5. Furthermore, the difference in the average kinetics of seeding using PD or DLB CSF may be indicative of a “strain” difference, similar to what has been observed from CSF samples from different types of CJD cases in prion RT-QuIC reactions [8, 25]. While the difference in the average kinetics is statistically significant (Welch’s t-test, *p* < 0.02) between the two seed types, at this point we do not know the basis for this difference, or whether it might be diagnostically useful. It could be due to differences in average seed concentration or seed characteristics, such as potential structural differences between PD and DLB seeds, that contribute to kinetic differences in the αSyn RT-QuIC responses. Testing of much higher numbers of PD and DLB samples is required to discern whether the difference in kinetics is maintained and if it is of diagnostic or mechanistic importance.

As our assay evolved, we saw evidence that several factors, such as sample volume, SDS concentration, temperature, and silica beads, strongly influenced the performance of the assay. However, at present we cannot be sure of the relative importance of these factors individually in allowing the assay to be substantially faster than previously described αSyn RT-QuIC or PMCA assays. Further testing will be necessary to better understand i) the breadth of synucleinopathies that are detected and/or discriminated by αSyn RT-QuIC assays, ii) the diagnostic sensitivities and specificities in clinical settings, iii) the extent to which levels of αSyn seeding activity in CSF or other diagnostic specimens correlates with disease prognosis, and iv) the relevance of αSyn^D^ seeding activity as a biomarker in therapeutic trials.

## Conclusions

Compared to previously described aSynD seed amplification assays, the use of the raSyn substrate preparation, RT-QuIC reaction conditions, and end-point dilution assays that we describe here allowed for much more rapid detection and quantitation of aSyn seeding activity in CSF of patients with PD and DLB without reductions in diagnostic sensitivity or specificity.

## Additional files

Additional file 1: Example of K23Q purification chromatograph, and total protein staining of collected fractions and of the commercial wild-type αSyn (WT^*^), and our preparation of the WT and K23Q rαSyn substrates. Absorbance spectra (blue) and buffer B gradients (green) for purification of K23Q using a Ni-NTA column (A and B) followed by a Q-HP column (C and D). Arrow in A denotes a peak representing contaminants. Dashed boxes in A and C denote the zoomed regions depicted in B and D. The * in C and D denote contaminants. The red vertical lines in B delineate the peak collected from the Ni-NTA column between 30 and 75% of buffer B1 (containing 100 and 350 mM imidazole, respectively) for further purification on the Q-HP column. The red lines in D indicate the peak collected from the Q-HP column between 30 and 35% of buffer B2 (containing 300 and 350 mM NaCl, respectively) to be used for dialysis and lyophilization. Panels E and F are a total protein Coomassie Blue staining of fractions collected from the Ni-NTA and Q-HP column, respectively. Panel G shows a comparative total protein Coomassie Blue staining of 2 μg of the commercial wild-type αSyn (WT^*^), and both of our WT and K23Q rαSyn substrate preparations. The staining intensity and apparent molecular weight of the WT^*^ differs from our prepared WT and K23Q due to the lack of a poly-histidine tag [37], therefore a 5-fold higher amount of WT^*^ was run in panel H to investigate for potential contaminants with a higher intensity staining. (TIFF 5283 kb)

Additional file 2: Detection of αSyn seeding activity in BH and CSF using K23Q (blue), WT (red) and WT* (green; commercial wild-type rαSyn lacking a 6× histidine tag [7]) substrates as described in Fig. 1 but with standard deviation. For clarity error bars are only displayed in one direction. (TIFF 1254 kb)

Additional file 3: Demographic and clinical information for subjects of this study. (XLSX 17 kb)

Additional file 4: αSyn RT-QuIC end-point dilution analysis of one Parkinson’s (PD; 081017) and three dementia with Lewy bodies (DLB; 2004–16, 2006–005 and 2006–020) brain samples listed in Fig. 4. Reactions were seeded in quadruplicate with two μl of either a 10^− 4^, 10^–5,^ 10^− 6^ or a 10^− 7^ brain homogenate (BH) dilutions. Each sample trace represents the average ThT signal of quadruplicate wells. (TIFF 852 kb)

Additional file 5: End-point dilutions by αSyn RT-QuIC of synucleinopathy CSF samples listed in Fig. 4. Each sample trace represents the average ThT signal of quadruplicate wells. Traces represent 15 (Blue), 7.5 (Red), 3.75 (Green), 1.89 (Purple), 0.94 (Orange), 0.47 (Black), 0.2 (Brown) and 0.1 (Dark Blue) μL of DLBD CSF diluted into normal pooled CSF, when needed, to give overall CSF sample volumes of 15 µL. (TIFF 1383 kb)
