# Correction to: Rapid and ultra-sensitive quantitation of disease-associated α-synuclein seeds in brain and cerebrospinal fluid by αSyn RT-QuIC

**DOI:** 10.1186/s40478-020-01052-y

**Published:** 2020-11-05

**Authors:** Bradley R. Groveman, Christina D. Orrù, Andrew G. Hughson, Lynne D. Raymond, Gianluigi Zanusso, Bernardino Ghetti, Katrina J. Campbell, Jiri Safar, Douglas Galasko, Byron Caughey

**Affiliations:** 1 Laboratory of Persistent Viral Diseases, Rocky Mountain Laboratories, National Institute of Allergy and Infectious Diseases, National Institutes of Health, Hamilton, MT USA; 2 Department of Neurosciences, Biomedicine and Movement Sciences, University of Verona, Verona, Italy; 3 Indiana University School of Medicine, Indianapolis, IN USA; 4 Department of Pathology, Case Western Reserve, University School of Medicine, Cleveland, OH USA; 5 Department of Neurosciences, University of California-San Diego, La Jolla, CA USA

## **Correction to:** Acta Neuropathologica Communications (2018) 6:7 10.1186/s40478-018-0508-2

In the original publication of this article [1] the plasmid name is incorrect in the first sentence of the “**K23Q rαSyn expression vector preparation”** section of **Materials and methods**.

The correct and incorrect information is shown below in **bold**.

- **Correct:** DNA sequences coding for human α-synuclein sequence (Accession No. NM_000345.3) amino acid residues 1–140 (wildtype) were amplified and ligated into the **pET28 vector** with an N-terminal His-tag (EMD Biosciences) and sequences were confirmed.
- **Incorrect:** DNA sequences coding for human α-synuclein sequence (Accession No. NM_000345.3) amino acid residues 1–140 (wildtype) were amplified and ligated into the **pET24 vector** with an N-terminal His-tag (EMD Biosciences) and sequences were confirmed.

Furthermore, the original publication contained an incorrrect version of figure 4. The error in this figure is: exponents in the SD50/mg column of panel A are negative, when they should be positive. This correction article contains the correct version of Fig. 4.

**Fig. 4:**
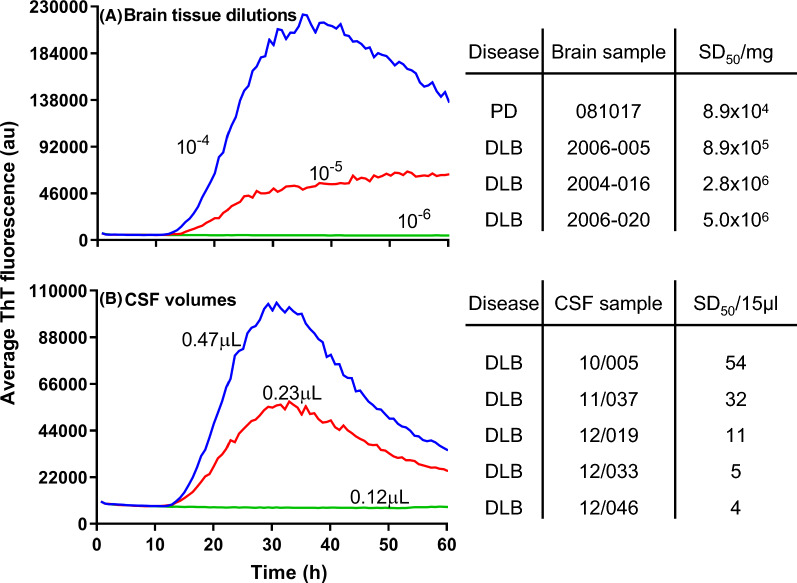
End-point dilutions of synucleinopathy BH (**a**; sample # 081017) or CSF (**b**; sample # 10/005) samples by αSyn RT-QuIC. Each sample trace represents the average ThT signal of quadruplicate wells. Tables to the right of each graph indicate the concentration of SD_50_ units calculated by Spearman–Kärber analysis for these, and additional, cases. End-point dilution experiments used for the additional calculated values shown in the upper and lower panels are provided in Additional files 4 and 5, respectively
