# Supplementary figures and images for: Rapid and ultra-sensitive quantitation of disease-associated α-synuclein seeds in brain and cerebrospinal fluid by αSyn RT-QuIC
Source: Acta Neuropathol Commun. 2018 Feb 9;6:7. doi: 10.1186/s40478-018-0508-2 (PMC5806364; doi:10.1186/s40478-018-0508-2)

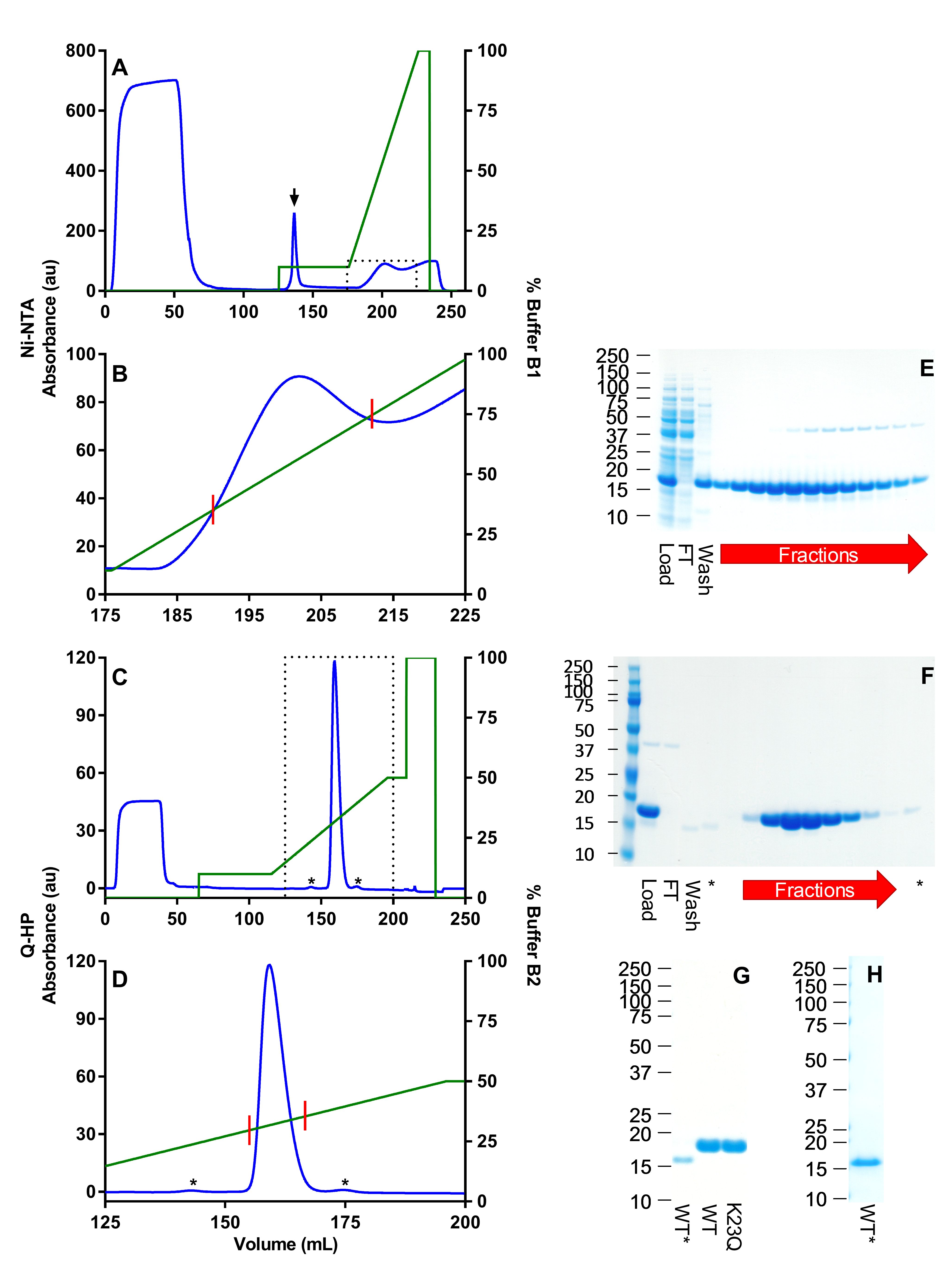

Supplement: Supplementary file 1 — Example of K23Q purification chromatograph, and total protein staining of collected fractions and of the commercial wild-type αSyn (WT*), and our preparation of the WT and K23Q rαSyn substrates. Absorbance spectra (blue) and buffer B gradients (green) for purification of K23Q using a Ni-NTA column (A and B) followed by a Q-HP column (C and D). Arrow in A denotes a peak representing contaminants. Dashed boxes in A and C denote the zoomed regions depicted in B and D. The * in C and D denote contaminants. The red vertical lines in B delineate the peak collected from the Ni-NTA column between 30 and 75% of buffer B1 (containing 100 and 350 mM imidazole, respectively) for further purification on the Q-HP column. The red lines in D indicate the peak collected from the Q-HP column between 30 and 35% of buffer B2 (containing 300 and 350 mM NaCl, respectively) to be used for dialysis and lyophilization. Panels E and F are a total protein Coomassie Blue staining of fractions collected from the Ni-NTA and Q-HP column, respectively. Panel G shows a comparative total protein Coomassie Blue staining of 2 μg of the commercial wild-type αSyn (WT*), and both of our WT and K23Q rαSyn substrate preparations. The staining intensity and apparent molecular weight of the WT* differs from our prepared WT and K23Q due to the lack of a poly-histidine tag [37], therefore a 5-fold higher amount of WT* was run in panel H to investigate for potential contaminants with a higher intensity staining. (TIFF 5283 kb) [file 40478_2018_508_MOESM1_ESM.tif]

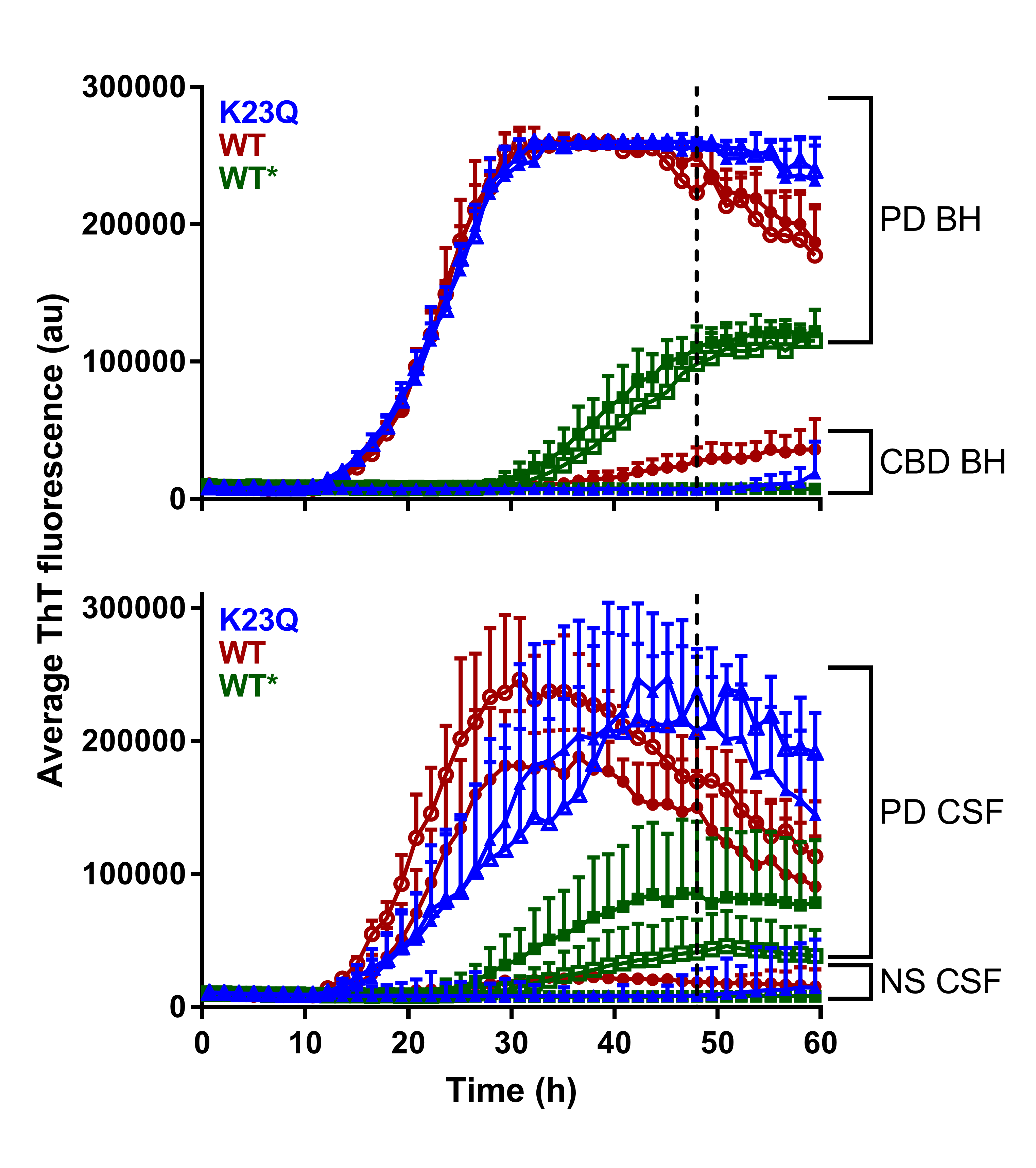

Supplement: Supplementary file 2 — Detection of αSyn seeding activity in BH and CSF using K23Q (blue), WT (red) and WT* (green; commercial wild-type rαSyn lacking a 6× histidine tag [7]) substrates as described in Fig. 1 but with standard deviation. For clarity error bars are only displayed in one direction. (TIFF 1254 kb) [file 40478_2018_508_MOESM2_ESM.tif]

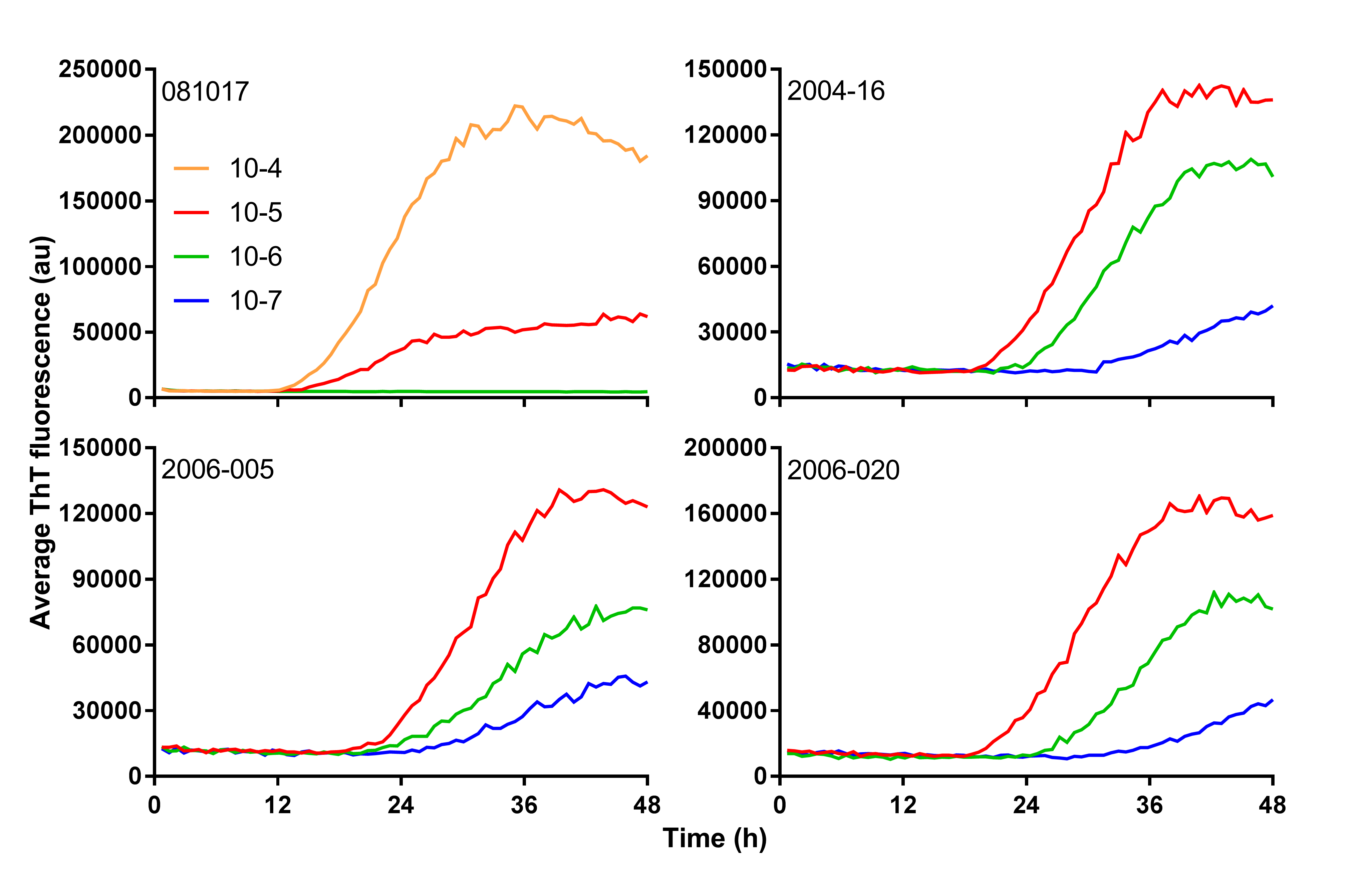

Supplement: Supplementary file 4 — αSyn RT-QuIC end-point dilution analysis of one Parkinson’s (PD; 081017) and three dementia with Lewy bodies (DLB; 2004–16, 2006–005 and 2006–020) brain samples listed in Fig. 4. Reactions were seeded in quadruplicate with two μl of either a 10− 4, 10–5, 10− 6 or a 10− 7 brain homogenate (BH) dilutions. Each sample trace represents the average ThT signal of quadruplicate wells. (TIFF 852 kb) [file 40478_2018_508_MOESM4_ESM.tif]

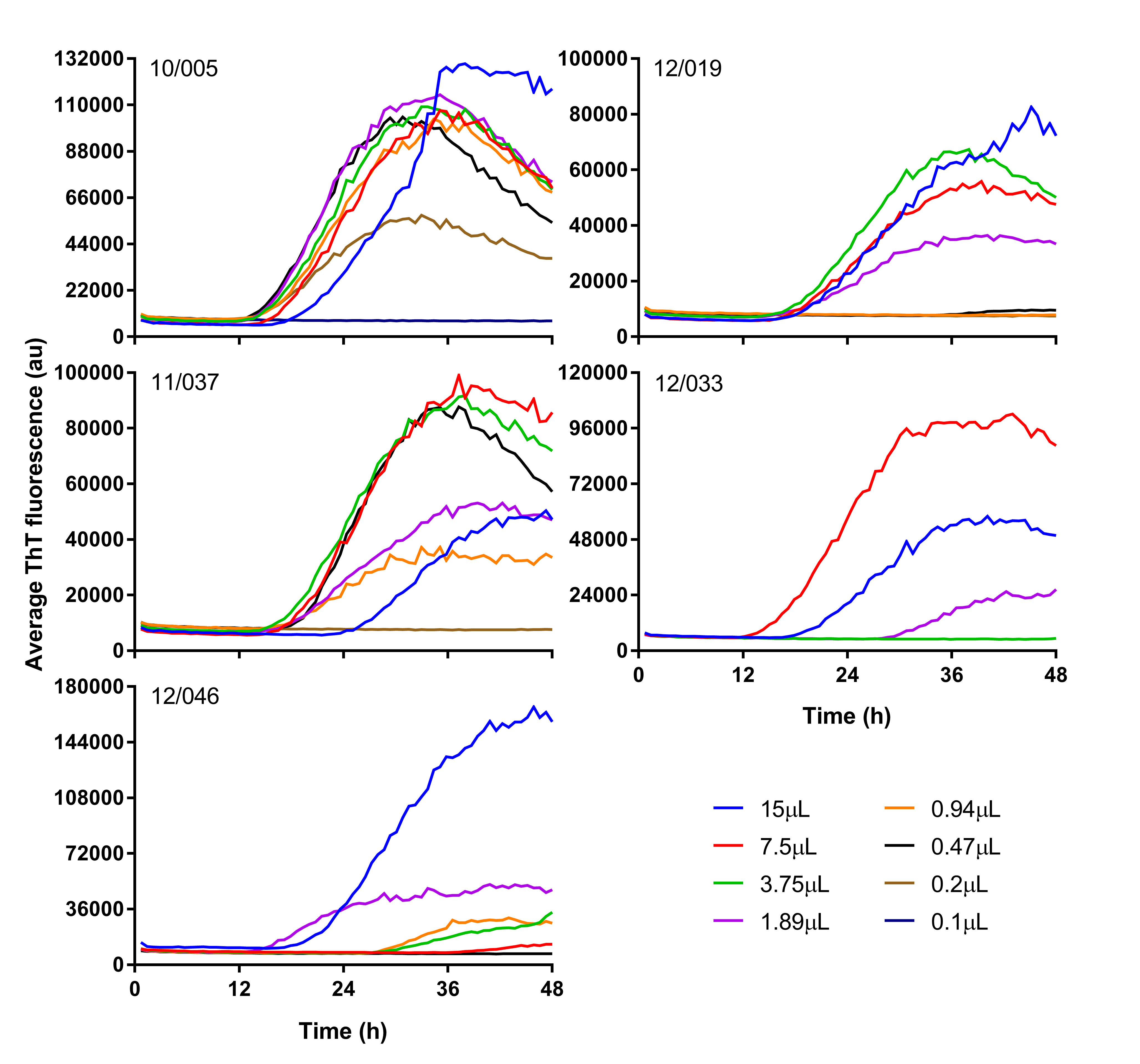

Supplement: Supplementary file 5 — End-point dilutions by αSyn RT-QuIC of synucleinopathy CSF samples listed in Fig. 4. Each sample trace represents the average ThT signal of quadruplicate wells. Traces represent 15 (Blue), 7.5 (Red), 3.75 (Green), 1.89 (Purple), 0.94 (Orange), 0.47 (Black), 0.2 (Brown) and 0.1 (Dark Blue) μL of DLBD CSF diluted into normal pooled CSF, when needed, to give overall CSF sample volumes of 15 µL. (TIFF 1383 kb) [file 40478_2018_508_MOESM5_ESM.tif]
